# Free-energy studies reveal a possible mechanism for oxidation-dependent inhibition of MGL

**DOI:** 10.1038/srep31046

**Published:** 2016-08-08

**Authors:** Laura Scalvini, Federica Vacondio, Michele Bassi, Daniele Pala, Alessio Lodola, Silvia Rivara, Kwang-Mook Jung, Daniele Piomelli, Marco Mor

**Affiliations:** 1 Dipartimento di Farmacia, Università degli Studi di Parma, I-43124 Parma, Italy; 2 Department of Anatomy and Neurobiology, University of California, Irvine, Irvine, CA 92697, United States; 3 Department of Biological Chemistry, University of California, Irvine, Irvine, CA 92697, United States; 4 Unit of Drug Discovery and Development, Istituto Italiano di Tecnologia, I-16163, Genova, Italy

## Abstract

The function of monoacylglycerol lipase (MGL), a key actor in the hydrolytic deactivation of the endocannabinoid 2-arachidonoyl-*sn*-glycerol (2AG), is tightly controlled by the cell’s redox state: oxidative signals such as hydrogen peroxide suppress MGL activity in a reversible manner through sulfenylation of the peroxidatic cysteines, C201 and C208. Here, using as a starting point the crystal structures of human MGL (hMGL), we present evidence from molecular dynamics and metadynamics simulations along with high-resolution mass spectrometry studies indicating that sulfenylation of C201 and C208 alters the conformational equilibrium of the membrane-associated lid domain of MGL to favour closed conformations of the enzyme that do not permit the entry of substrate into the active site.

Monoacylglycerol lipase (MGL), a presynaptic serine-hydrolase of the α/β hydrolases superfamily, terminates the biological actions of the major lipid neurotransmitter found in the CNS, the endocannabinoid 2-arachidonoyl-*sn*-glycerol (2-AG)^1^,^2^. As other lipid hydrolases^3^,^4^,^5^,^6^, MGL captures its substrate from the cellular membrane via a flexible region known as lid domain. This structural element, which in human MGL (hMGL) comprises a U-shaped funnel delimited by the helix α4 and the loop between helices α4 and α5^7^,^8^,^9^,^10^,^11^, serves as a flexible gate that allows access of 2-AG to the enzyme’s catalytic region (Fig. 1). While the lid domain of MGL is markedly hydrophobic, experimental data showing that the enzyme is equally distributed between cytosol and membrane fractions suggest that MGL is an amphitropic protein that can reside either in the cytosol or on cell membranes, where it is most likely to encounter its lipophilic substrate 2-AG^1^. Closely inserted within the lid domain, two cysteine residues, C201 and C208, have been recently found to play a key role in modulating the activity of both rat and human MGL^12^,^13^. In particular, experiments in rats have shown that MGL undergoes an oxidation-dependent form of activity regulation that depends on the reversible conversion of the cystenyl groups of C201 and C208 into sulfenic acid^14^. A third cysteine residue, C242, situated in close contact with the catalytic triad (S122, H269, D239), directly regulates enzymatic activity^13^,^15^ and is not involved in this regulatory process^14^ (Fig. 1).

Recent work has provided evidence that the interaction between hMGL and models of biological membranes, mimicking the transition from a cytosolic form of the enzyme to a membrane-bound one, cause major rearrangements of the lid-domain conformation^16^. Notably, such rearrangements are associated with an improvement of the enzyme kinetics parameters, suggesting that MGL, similarly to other lipases, is subject to interfacial activation^17^,^18^,^19^. On the other hand, NMR experiments have revealed that the cytosolic form of hMGL lives in equilibrium between an active and an inactive form^20^, and that this equilibrium might be governed by conformational transitions of the lid domain from an open, active site-accessible state, to a closed and inactive one.

Crystallographic coordinates of rMGL are not available, whereas the structure of hMGL has been resolved in different conformations^11^. Specifically, the available three-dimensional structures of the enzyme present the lid domain in two different conformations, characterized by a closed gate over the catalytic triad (3PE6) or, conversely, by an open active site-access channel (3HJU). A further crystallographic structure (3JW8) is characterized by an open lid domain state, with the helix α4 adopting an intermediate conformation between those assumed by 3PE6 and 3HJU. Superposition of the different crystal structures (Fig. 2a) thus outlines that the lid domain exhibits a conformational equilibrium, where different conformations could be favoured by the presence of inhibitors (3PE6) or, on the contrary, by specific treatments during the crystallization protocol, such the use of surfactants (3HJU and 3JW8).

The lid-domain conformational space has been widely investigated in the last decade, and the results of different molecular modelling studies of the human^21^ and of the bacterial form of the enzyme^22^ consistently reveal that the lid domain of MGL spontaneously undergoes wide conformational modifications.

Starting from the reasonable assumption that the conformational spaces of the rat and human enzymes are sufficiently similar, since the two proteins show >80% sequence homology, we used available crystal structures of hMGL to build membrane-associated models and to explore the effect of reversible oxidation of C201 and C208 on the conformational equilibria of the lid domain.

## Results

### hMGL activity depends on the redox state of its environment

Recent data provide strong evidence for a role of C201 and, to a lesser extent, C208 as activity switchers for rMGL, which they regulate through the reversible formation of sulfenylated forms^14^. To investigate whether a similar mechanism governs the activity of hMGL, we incubated recombinant, commercial hMGL in buffered solutions containing various redox reagents. Dithiothreitol (DTT), which is commonly used to prevent the oxidation of cysteine residues of proteins to disulfides or to other reversibly oxidized species (i.e. sulfenic acids; sulfenamides)^23^, activated hMGL in a concentration-dependent manner (Fig. 3a) such that, at DTT concentrations of 1 mM, hMGL activity was more than five-fold higher than that of control. A similar, albeit less marked, effect was observed with glutathione and sodium sulphite, which enhanced hMGL activity with median effective concentrations (EC_50_) values of approximately 0.1 mM and 0.05 mM, respectively. (Fig. 3b,c). Conversely, incubation of hMGL with hydrogen peroxide (H_2_O_2_) caused a decrease in hMGL activity, as previously reported for rMGL^14^. The inhibitory potency of H_2_O_2_ on hMGL was, however, less pronounced than that measured with rMGL (EC_50_ = 10 mM, Supplementary Fig. S1). Given the marked sensitivity of commercial hMGL to activation by reducing agents, it is reasonable to conclude that the redox equilibrium of this enzyme preparation is shifted towards a more oxidized and catalytically less active state.

### Hydrogen peroxide sulfenylates C201

hMGL contains four cysteine residues (C32, C201, C208, C242), which could all in principle undergo oxidative modification. In rMGL, which contains the same residues, only C201 and C208 are oxidized by H_2_O_2_^14^. To identify the residue(s) targeted by this oxidant in hMGL, we used the sulfenic acid-selective probe, dimedone (DMD). DMD is a cyclic diketone that (i) selectively binds to the labile cysteine sulfenic acids, forming stable thioethers that are not cleavable by reducing reagents, such as DTT, and can be detected by mass spectrometry (MS) and (ii) does not react with protein thiols, disulfides, nitrosothiols, sulfenamides, sulfinic and sulfonic acids^24^. We used commercial hMGL both in its native state and after treatment with H_2_O_2_. As shown in Fig. 3d, incubation of hMGL with DMD followed by reduction with DTT led to approximately the same 5-fold increase in hMGL activity as observed with incubation with DTT alone. After the treatment of hMGL with H_2_O_2_, DTT still increased enzyme activity, but addition of DMD during pre-incubation blocked activation by DTT. This behaviour was observed on different hMGL batches. The results show that treatment with H_2_O_2_ yields a reversibly oxidized form of hMGL that can be irreversibly inhibited by DMD.

To identify the cysteine residues that react with DMD, we incubated hMGL with H_2_O_2_ in the presence of DMD, digested the protein overnight with trypsin, and subjected the lysate to peptide mass fingerprint analysis by MALDI-TOF/TOF MS, after reduction with DTT and alkylation with iodoacetamide. The protocol yielded a 71% of sequence coverage of MGLL_HUMAN (Mascot Score: 88) with C32-, C201- and C208-containing peptides represented with good mass accuracy (Δm < 6 ppm) and signal-to-noise (S/N) ratios (>90) (Table 1). A C242-containing peptide was also retrieved, but with a very low S/N ratio. These peptides had already been attributed by MALDI-TOF MS fingerprinting of a recombinant hexa-histidine-tagged hMGL^25^. After incubation of a second aliquot of hMGL with H_2_O_2_ and DMD, followed by the same reduction/alkylation/trypsin digestion protocol, identical peptides of control hMGL were retrieved in MS Mascot analysis. Moreover, two peptides at *m/z* = 2018.0078 ([M+H]^+^) and *m/z* = 1775.8528 ([M+H]^+^) were retrieved corresponding to two C201-containing peptides (aa 187–202; aa 189–202) bound to dimedone (Table 2 and Fig. 4). Subsequent MALDI-TOF/TOF analysis of the C201-containing peptide NKTEVDIYNSDPLICR (aa 187–202) allowed us to identify one abundant fragment, corresponding to the y-5 fragment at the N-terminus of the proline residue. This fragment, of sequence PLICR, included C201 in its reduced state or with the expected modifications, either IAA or DMD (Table 3 and Supplementary Fig. S2).

Thus, despite the low S/N ratios of the corresponding DMD-modified peptides, it was possible to detect the site of DMD covalent modification at C201. This result confirmed that, also in human MGL, C201 is sensitive to reversible oxidation to sulfenic acid. Conversely, from our data, it was not possible to exclude, in H_2_O_2_-treated hMGL, the formation of an adduct with DMD for C242, which is found in close contact with the catalytic triad. C242 was not efficiently mapped by the trypsin digestion and MALDI-TOF detection protocol, due to the very low S/N ratio of the corresponding peptide in the tryptic peptide mixture. As DMD addition was shown to significantly lower the ion intensity of the corresponding peptide, the signal of the C242-DMD peptide may have dropped below the detection limit of the MALDI MS method. According to our data, C32, situated in a solvent-exposed cleft of the enzyme α/β core, and C208 could have been oxidized by H_2_O_2_ to sulfenic acids, but to a much lower extent, as the ion intensities of C32-, C201- and C208-carbamidomethylated peptides were similar in all tested samples, indicating a comparable ionization efficiency, but only DMD-modified C201-containing peptides were detected by mass peptide fingerprint analysis.

### Molecular modelling

Collectively, the available data suggest that rMGL and hMGL live in equilibrium between an active and an inactive form, and that this equilibrium can be shifted towards inactivation by spontaneous or H_2_O_2_-induced oxidation. Moreover, the data suggest that sulfenylation of C201 and, to a minor extent, C208, is responsible for this shift. This raises the intriguing question of how an ostensibly small chemical modification (conversion of sulfihydryl to sulfenyl), that is located at a significant distance from the catalytic site, can induce such as profound change in enzyme activity. To address this question, we performed unbiased Molecular Dynamics (MD) simulations coupled to enhanced sampling techniques, including the well-tempered (WT) implementation of Metadynamics^26^,^27^, using the crystallographic coordinates of hMGL^8^.

### hMGL prefers a closed conformation in water

**Figure 2:**
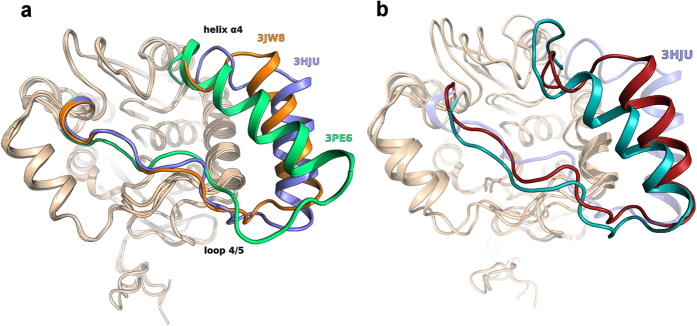
Comparison of available crystal structures, characterized by different lid domain conformations (**a**). 3HJU (light blue cartoon) is characterized by a widely open lid-domain conformation, while in 3PE6 (green cartoon) a rotational movement of helix α4 determines the closure of the active site access channel. The crystal structure 3JW8 (orange cartoon) reveals an intermediate conformation. (**b**) Comparison of two representative final conformations from 60-ns MD simulations of MGL in water (dark green cartoon) and in the presence of a membrane model (dark red cartoon), starting from the conformation of the crystal structure 3HJU (light blue cartoon).

**Figure 3:**
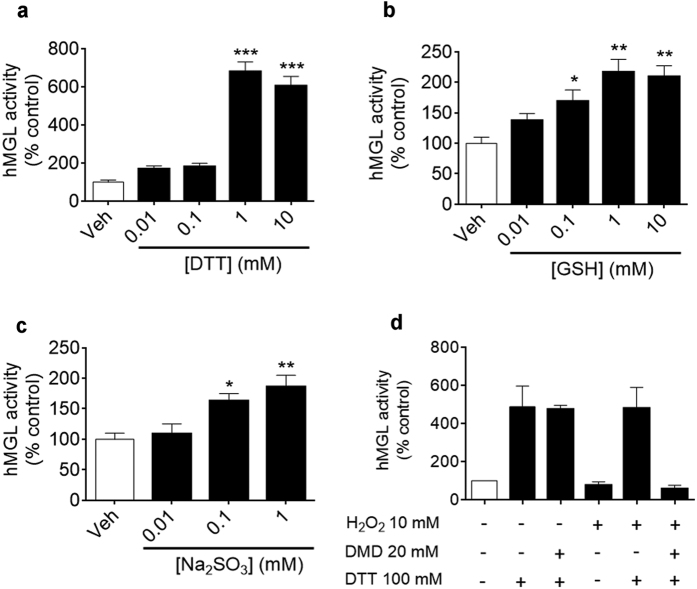
(**a**–**c**) Effects of increasing concentrations of DTT (**a**) GSH (**b**) and sodium sulphite (**c**) on hMGL activity (filled bars). hMGL activity in the presence of vehicle (open bars) was set as 100%. Error bars represent the SD (n = 3). ***P < 0.001, **P < 0.005 and *P < 0.05 compared with vehicle, one-way ANOVA. (**d**) Effect of H2O2, DTT and DMD on hMGL basal activity. hMGL activity in the presence of vehicle was set as 100%. Error bars represent the SD (n = 3).

**Figure 4:**
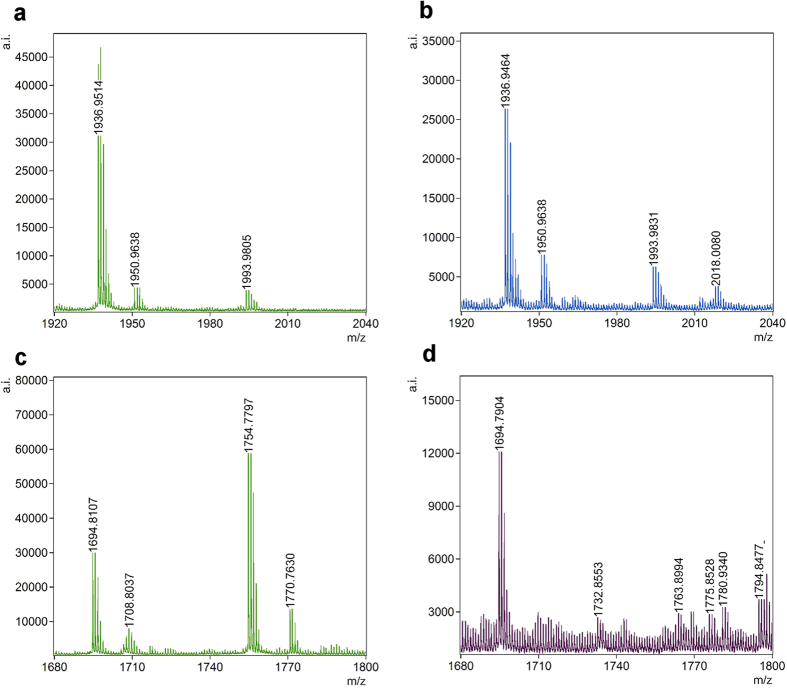
(**a**) MALDI-TOF spectrum of CAM-modified (*m/z* = 1936.9575; [M+H]^+^) C201-containing peptide 187–202 in control hMGL. (**b**) MALDI-TOF spectrum of CAM- (*m/z* = 1936.9464; [M+H]^+^) and DMD-modified (*m/z* = 2018.0080; [M+H]^+^) C201-containing peptide 187–202 in H_2_O_2_-treated hMGL. (**c**) MALDI-TOF spectrum of CAM-modified (*m/z* = 1694.8107; [M+H]^+^) C201-containing peptide 189–202 in control hMGL. (**d**) MALDI-TOF spectrum of CAM- (*m/z* = 1694.7904; [M+H]^+^) and DMD-modified (*m/z* = 1775.8528; [M+H]^+^) C201-containing peptide 189–202 in H_2_O_2_-treated hMGL.

We first ran two sets of six 60-ns MD simulations of native hMGL, starting from its open lid-domain conformation (PDB: 3HJU)^8^, and of the enzyme modelled with C201 oxidized to sulfenic acid, using water as explicit solvent. Analysis of root mean square fluctuation (RMSF) of the protein backbone showed that the α/β core of the protein, including the region of the catalytic triad, had a high degree of dynamical stability, indicating that modification of the C201 side chain had no influence on the architecture of the active site. Conversely, the lid domain region revealed a high flexibility and showed large structural fluctuations that involved helix α4 and the loop connecting helix α4 to helix α5 (Supplementary Fig. S3). The observed conformational rearrangement eventually consisted in an approaching movement of the lid domain rims, leading to the closure of the gate over the active site access. The lid domain movements were analysed by measuring the variation of the distances between the centres of mass of selected residues carbon α (Cα). The lid domain-closing event was observed with variable frequency, depending on the model considered. Specifically, the lid domain of native hMGL was characterized by a high flexibility and showed, in two out of six simulations, a complete closure of the aperture over the active site, with the helix α4 assuming a conformation comparable to that of the crystal structure 3PE6 (Fig. 2b). In two other simulations the distances used as a parameter to evaluate the extent of lid domain opening increased, unveiling a spontaneous transition from an open to a more open state in aqueous solvent. Conversely, within the set of MD simulations performed on hMGL modelled with C201 mutated to sulfenic acid, the lid domain-closing event was observed in four simulations out of six.

In order to provide information about the free-energy changes involved in the lid domain closure mechanism, we performed WT Metadynamics studies on native hMGL, using the distances between the centres of mass of Cα of residues lining the lid domain rims as collective variables (CV1 and CV2, respectively). In order to set a reliable parameter to discriminate between the open and closed lid domain conformations, we considered the CVs values corresponding to the distances measured from the MD simulations described above (See Methods for definition of CV1 and CV2). Specifically, low CVs values correspond to a reduced distance between the lid domain rims, indicating the closure of the active site access funnel.

The free-energy surface (FES) profile showed the presence of an energy well corresponding to a closed conformation, as a consequence of the reduction of the distance between the lid domain edges along the CV1 and, at a lower extent, the CV2. Interestingly, we could not determine a free-energy minimum corresponding to the open lid domain conformation, that is, the initial conformation adopted by the enzyme as obtained from the crystal structure 3HJU. These results, obtained from classical MD simulations and from free-energy studies, are in accordance with the phenomenon of interfacial activation. In this view, the marked hydrophobic nature of hMGL lid domain might have affected the exploration of the enzyme conformational space in a polar environment. Furthermore, the crystal structure used in this work was obtained in presence of surfactants, which may have influenced the architecture of the lid domain, promoting its open conformation.

### The membrane promotes the opening of hMGL lipid gate

To evaluate the effect of the interaction between hMGL and cell membranes on the conformational equilibrium of the lid domain, we performed a molecular dynamics simulation of the enzyme in the presence of a POPC phospholipid model. Contrary to what has been observed for the MD simulation of hMGL in water, the enzyme embedded in the membrane showed the tendency to maintain an open lid-domain state, with the helix α4 adopting a conformation comparable to that observed in the crystal structure 3JW8 (Fig. 2b).

In order to further investigate the role of the membrane in the mechanism ruling the open to close transition of the lid domain, we performed WT Metadynamics studies in the presence of a POPC phospholipid model, using the same CV set used in Metadynamics simulations of hMGL in water.

**Figure 5:**
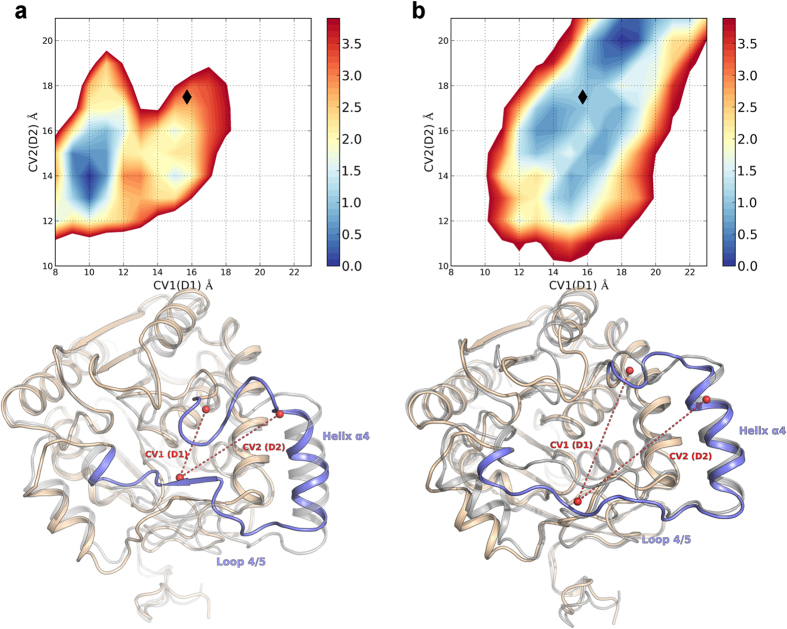
Comparison of the FES obtained for native hMGL in water (**a**) and in presence of a model membrane (**b**). Black diamonds mark the CVs values measured for the crystal structure of hMGL in its open conformation (PDB 3HJU, grey cartoon). For the free-energy minima, representative structures are reported; the lid domain portion corresponding to helix α4 and loop 4/5 is depicted in light blue. The CVs values extent is represented by red dashes. The structures from WT Metadynamics simulations are superposed to the crystal structure of hMGL in its open conformation (grey cartoon).

In this case, the FES obtained showed notable differences in comparison to the free-energy profile of hMGL in water. A free-energy minimum corresponding to the open lid domain conformation was present and, more interestingly, the FES profile disclosed the tendency of the system to adopt an open conformation, characterized by a wider aperture over the active site (Fig. 5). Conversely, the FES did not show free-energy well corresponding to the closed lid domain (Fig. 5). Collectively, these computational analyses support the hypothesis that MGL regulates the 2-AG engagement through the interaction with the phospholipid bilayer, which in turn promotes substrate recruitment by enhancing the active site access disclosure.

### Free-energy studies of hMGL modelled with sulfenylated C201 and with a single point mutation C201A in presence of membrane

The free-energy studies outlined above suggest that the presence of a phospholipid bilayer stabilizes hMGL in an open conformation, which is energetically disfavoured in water. We hypothesized that oxidation of C201 to sulfenic acid may be sufficient to perturb this conformational equilibrium and induce a closure of the lid domain. To test this idea, we performed WT Metadynamics simulations of hMGL modelled with C201 oxidized to sulfenic acid. As a control, we conducted parallel analyses on hMGL modelled with C201 mutated into alanine. We selected this mutation because previous studies have shown that single-point mutation of C201 or C208 to alanine does not affect the kinetics of hMGL or rMGL^14^,^28^. We reasoned that this comparison would allow us to assess the ability of our computational model to discriminate between inactivating (i.e. sulfenylation) and neutral (mutation to alanine) chemical modifications of C201.

**Figure 6:**
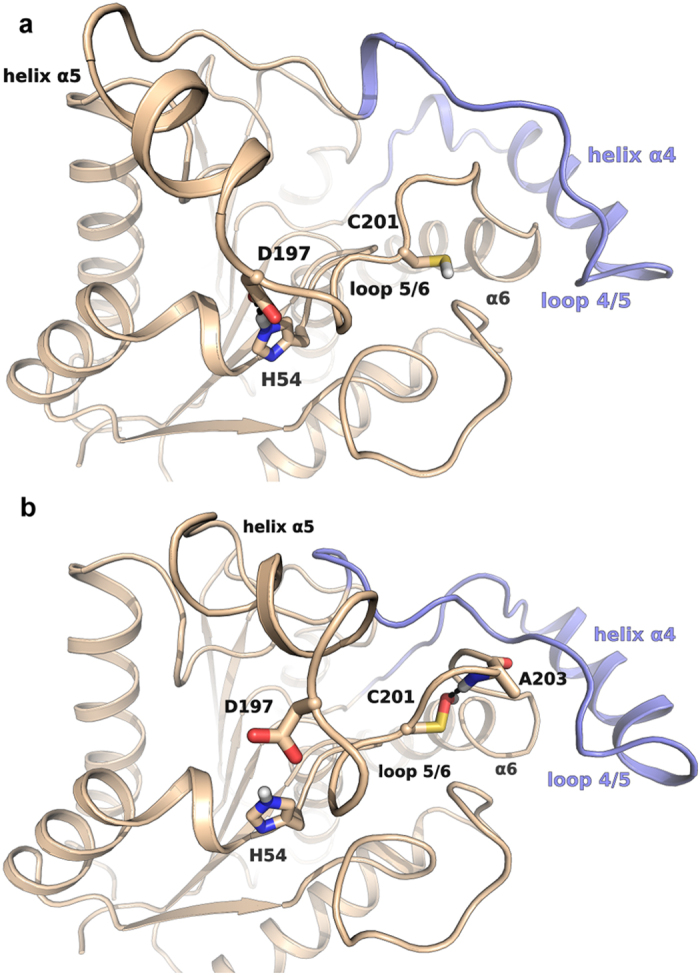
The open lid-domain conformation is favoured by the maintenance of the polar interaction between H54 and D197 (**a**). The modification of C201 into sulfenic acid promotes a conformational rearrangement of the loop 5/6 (**b**). This event is related to the formation of a hydrogen bond between the sulfenyl function of oxidized C201 with the backbone of A203 and to the disruption of the polar contact between H54 and D197, which bring to the reshaping of the overhead loop 4/5.

Despite the stabilizing effect of a model membrane, the FES of hMGL modelled with C201 mutated to sulfenic acid was remarkably different from the FES obtained for native hMGL, showing an opposite tendency of the system to populate a closed-lid domain conformation of the phase space. Specifically, the FES presented an energy minimum corresponding to a conformation characterized by a marked reduction in access to the active site. This rearranging event appeared to be mainly driven by the reduction of CV2 and, at a lower extent, of CV1, and was completely opposite to the tendency of native hMGL to adopt a conformation characterized by a wider opening of the lid domain funnel. Notably, the closure event involved a wide reshaping of the loop connecting the helices α5 and α6 (loop 5/6), where C201 is located. The modification of C201 to a sulfenic acid allowed the formation of a stable polar interaction between the oxygen atom and the backbone nitrogen of A203 (Fig. 6), determining a concerted conformational rearrangement of the loop 5/6 and of the overhead loop 4/5. Deformation of the loop 5/6, which determined the closure of the lid domain, also caused the disruption of a polar interaction between the residues H54 and D197, which was conversely maintained throughout all the simulations of the enzyme showing an open lid domain, e.g. those of the wild-type enzyme with the membrane model (Fig. 6). Recent NMR experiments have provided evidences that breaking of this polar interaction is associated to an inactive state of the enzyme, which has been attributed to shifting of the conformational balance towards a closed lid-domain state^20^. This supports the results of our simulations, confirming that MGL modulation is driven by a concerted regulation of the lid domain conformational equilibrium, which is affected by several factors, including the interaction with the membrane and, as here proposed, the change of regulatory cysteines redox state.

**Figure 7:**
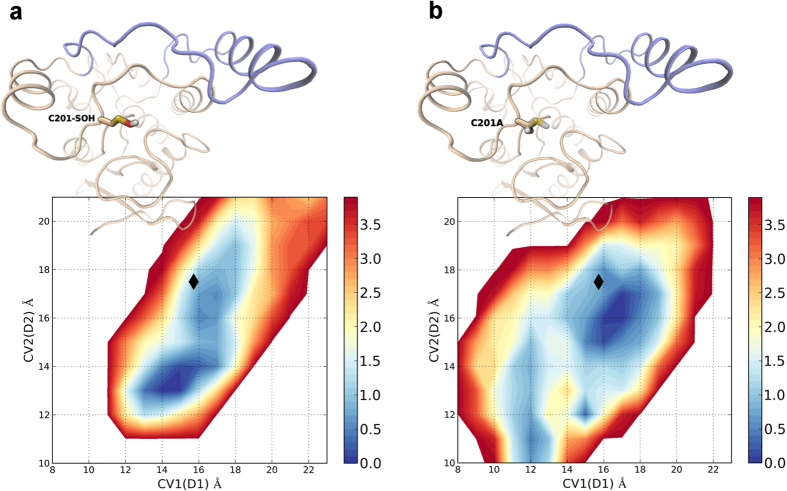
Comparison of the FES profile of hMGL modeled with C201 oxidized to sulfenic acid (**a**) and with C201 mutated to alanine (**b**). Black diamonds mark the CVs values measured for the crystal structure of hMGL in its open conformation.

Notably, the FES profile obtained for hMGL modelled with the single point mutation C201A confirmed the validity of our computational model, showing the presence of an energy basin corresponding to the open conformation. The FES indeed outlined the presence of free energy minima associated to slightly closed conformation of the lid domain; however the FES profile clearly showed the presence of an energy well matching the open conformation of the crystal structure 3HJU, which was completely absent on the FES of hMGL modelled with the oxidized cysteine (Fig. 7).

### Free-energy studies of hMGL with different modifications of C208 side chain

Encouraged by the results obtained from the free-energy studies on C201-modified hMGL, and asked whether the modification of C208, which is important in rMGL modulation, mediates conformational changes that can be related to the oxidation-dependent deactivation of the enzyme.

We thus prompted a model of hMGL with C208 mutated to alanine, in order to further test the validity of our computational protocol, and several models of hMGL with oxidized forms of C208. Specifically, we modelled hMGL with C208 and with both C201 and C208 transformed to sulfenic acid. Interestingly, while both the peroxidatic cysteines are fundamental to mediate the oxidation-driven MGL inhibition, the experimental data reveal that C208 is less prone than C201 to give a covalent adduct with DMD, suggesting that the sulfenic acid is not the main oxidized form responsible for C208-dependent blockade. C208 and the following residue, F209, are situated at the tip of helix α6, assuming a geometry that may favour, under oxidative conditions, a chemical reaction between the cysteine thiol group and the backbone nitrogen of F209, giving an internal sulfenamide. This transient oxidized form has been observed in the crystal structure of peroxiredoxin 6, where it is formed during storage^29^. It is quite unstable^30^,^31^,^32^,^33^, which can account for the reversible oxidation-dependent enzyme MGL inhibition and makes its detection a difficult task. While no experimental evidence for the formation of a sulfenamide is still available, we also tested the hypothesis that formation of a cyclic sulfenamide between C208 and the following residue F209 could affect the conformational equilibrium of the lid domain, leading to MGL inactivation.

Consistently with mutagenesis data, the FES obtained from the WT Metadynamics of hMGL with C208A mutation presented free-energy minima corresponding to open lid domain conformations, matching the active state conformation adopted by the initial crystal structure. Conversely, the modification of C208 to oxidized forms, including the oxidation of C208 or of both C201 and C208 to sulfenic acid, and the engagement of C208 thiol group in a sulfenamide, determined remarkable changes on the FES profile. Specifically, the FES profiles of hMGL with C208 and both C201 and C208 transformed into sulfenic acid presented free-energy minima corresponding to closed conformation, with the lid domain-closing event driven by the concurrent reduction of CV1 and CV2.

**Figure 8:**
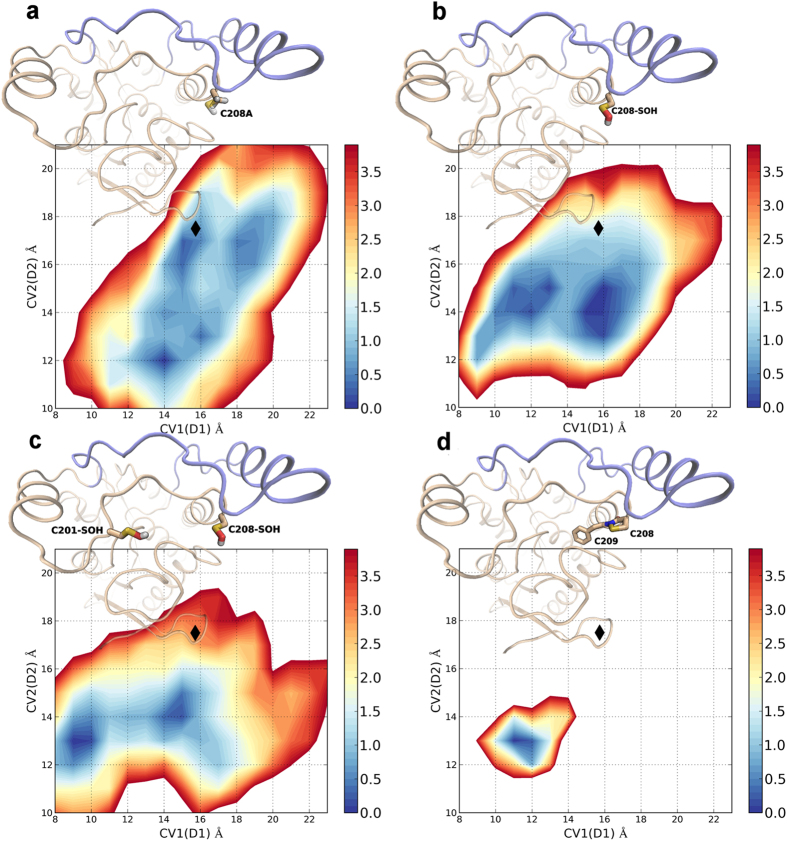
Comparison of the FES profiles of hMGL modelled with C208 mutated to alanine (**a**), with C208 oxidized to sulfenic acid (**b**), with both C201 and C208 oxidized to sulfenic acid (**c**), and with C208 and F209 involved in the formation of a sulfenamide. Black diamonds mark the CVs values measured for the crystal structure of hMGL in its open conformation (**d**).

Interestingly, the FES of hMGL with C208 and F209 involved in the formation of an internal sulfenamide showed an even deeper minimum, circumscribed in a region defined by a strong reduction of the active site access channel extent (Fig. 8).

## Discussion

The results of our study show that, similarly to what has been observed for the rat form of MGL^14^, activity of the human enzyme is higher under reductive conditions, and that its cysteine residue C201 can be reversibly oxidized to sulfenic acid. Our molecular simulations offer a plausible explanation for MGL inhibition by oxidative stimuli mediated by the regulatory cysteines, C201 and C208, located within the enzyme lid domain. Previous experimental and computational studies have proposed the existence of different conformations for MGL^20^ and the influence of biological membranes on lid-domain conformational equilibrium^16^. Our simulations, and in particular the free-energy surfaces calculated by metadynamics simulations with two collective variables related to active-site accessibility, provide a mechanistic model of interfacial activation for MGL. In fact, while in a water environment a closed conformation of the lid domain results favoured, simulations within a model membrane show a free-energy minimum for wild-type hMGL corresponding to an open conformation, with free access to the catalytic site for a substrate molecule. Our computational protocol, supported by its consistence with NMR and mutagenesis data, predicts that oxidation of C201 and/or C208 to sulfenic acid alters the conformational equilibrium of the lid domain, favouring the closed form, even in the presence of the model membrane. Previous work on rat MGL, and results presented here on hMGL, suggest that C201-sulfenic acid is the chemical species responsible for enzyme inactivation by oxidative stimuli. On the other hand, the evidence supporting such a role for C208 is not definitive. In this context, our simulations suggest that formation of another oxidized species, a cyclic sulfenamide between C208 and the preceding residue, could play a role in MGL inactivation. In conclusion, this work provides a mechanistic hypothesis to explain the role of regulatory cysteine residues, located within the lid domain of MGL, on its inhibition by oxidation. These cysteines may act as chemical switches that, transformed to sulfenic acid or sulfenamide by oxidative stimuli produced in proximity of the membrane, affect interfacial activation of MGL and hamper the access of its substrate, 2-AG, from the membrane to the catalytic site. This is the first case of computational investigation on the molecular machinery linking oxidative chemical switch to interfacial activation of a lipase.

## Methods

### Materials

5,5-Dimethyl-1,3-cyclohexanedione (DMD), DMSO, chymotrypsin, dithiothreitol (DTT), heptadecanoic acid (HDA), iodoacetamide (IAA), 2-oleoyl-sn-glycerol (2-OG), H_2_O_2_, fatty acid-free BSA, trifluoroacetic acid (TFA) were purchased from Sigma-Aldrich. MS-grade trypsin “Trypsin Gold” was from Promega. Recombinant human MGL (hMGL) expressed in E. coli with an N-terminal Met and a 6His-tag was purchased from R&D Systems and it was supplied as a 0.411 μg/μl filtered solution in 25 mM Tris buffer pH 8.0 containing 1 M NaCl, 1 mM EDTA and 0.02% v/v Brij 35 as surfactant.

### Incubation of hMGL with Different Redox Reagents

*In vitro* hMGL activity was measured as described in Dotsey *et al.*^14^ with minor modifications. Briefly, recombinant hMGL (100 ng/sample) was pre-incubated for 10 min at 37 °C in 50 mM Tris-HCl buffer [pH 8.0] containing 0.5 mg/ml fatty acid-free BSA with (a) 100 mM DTT; (b) 20 mM DMD + 100 mM DTT; (c) 10 mM H_2_O_2_; (d) 10 mM H_2_O_2_ + 100 mM DTT; (e) 10 mM H_2_O_2_ + 20 mM DMD; (f) 10 mM H_2_O_2_ + 20 mM DMD + 100 mM DTT. The enzyme substrate 2-OG (100 μM) was then added and incubation at 37 °C continued for further 15 min. Enzymatic reaction was stopped by adding two vol of a chloroform/methanol mixture (1:1 vol/vol) containing HDA (5 nmol/sample) as internal standard for LC-MS/MS quantification. After centrifugation (2000 × g, 4 °C, 10 min), the organic layers were collected and dried under N_2_ flow. Samples were reconstituted in methanol and analysed by HPLC-MS/MS.

### HPLC-MS/MS analysis of oleic acid

Oleic acid, hydrolysis product of 2-OG, was analysed in negative ion mode (NIM) employing a TSQ Quantum Access MAX triple quadrupole mass spectrometer (Thermo) equipped with a heated electrospray ion source (H-ESI). The following parent-product ion transitions were selected: oleic acid: m/z 281.2 → 281.2 ([M-H]-; Tube Lens 78 V; Collision Energy: 5 eV), HDA (IS): m/z 269.2 → 269.2 ([M-H]-; Tube Lens 78 V; Collision Energy: 5 eV). A Phenomenex Synergi Fusion C18 column (100 × 2.0 mM, 4 μm) and the following gradient conditions were employed: A, 0.1% ammonium hydroxide in water; B, 0.1% ammonium hydroxide in methanol (B) at a flow rate of 350 μl/min. After 1 min at 60%A, a linear gradient was applied to 5%A in 7 min; then, after further 3 min at 5%A, it returned to 60%A, followed by 3 min reconditioning. Total run time was 14 min. H-ESI parameters were set as follows: probe middle (D) position; capillary temperature: 270 °C; ion spray voltage: −2.5 kV, vaporizer temperature: 250 °C. Nitrogen was used as nebulizing gas at the following pressure: sheath gas: 35 psi; auxiliary gas: 15 arbitrary units (a.u.). Argon was used as the collision gas at a pressure of approximately 1.5 mtorr (1 torr = 133.3 Pa). Data acquisition and processing were performed using Thermo Xcalibur software (version 2.1).

### Mass peptide fingerprint of native and oxidised hMGL

Human MGL (1.5 μM) was incubated in 50 mM Tris-HCl buffer [pH 8.0] in the presence of (i) vehicle or (ii) 10 mM H_2_O_2_ and 20 mM DMD for 60 min at 37 °C. Samples underwent a polyacrylamide gel electrophoretic run to get rid of the low MW reactants in excess. Gel was rinsed with Milli-Q water, bands of interest were excised and destained with a solution of ethanol/acetic acid/water (ratio 50:10:40 vol/vol/vol). Gel particles were then washed with water and acetonitrile, the liquid was removed and the gel particles were swollen in 50 mM ammonium bicarbonate [pH 8.0]. Reduction and alkylation of free cysteine residues occurred employing sequentially (i) 10 mM DTT for 30 min at 60 °C and (ii) 55 mM IAA for 20 min at room temperature in the dark. Then buffer was removed, the gel pieces were washed with new buffer and acetonitrile, dried under a N_2_ flow and rehydrated with 25 mM ammonium bicarbonate, 5 mM CaCl_2_ containing 12.5 ng/μl of MS-grade trypsin for 15 min at 4 °C. 5–25 μl of the same buffer without the enzyme were added to cover the gel pieces and samples were incubated overnight at 37 °C. To extract the tryptic peptides, the gel particles were added of acetonitrile and incubated for 15 min at 37 °C. The supernatant was collected. Then 25 μl of a 5% formic acid solution were added, replaced by acetonitrile and all supernatants were dried down under a N_2_ flow. Tryptic peptides were dissolved in 10–15 μl of 0.1% vol/vol TFA in water. ZipTip C_18_ purification was performed before mixing the samples with MALDI matrix HCCA (5 mg/ml in 60% acetonitrile/0.1% TFA). 1 μL was spotted onto an Opti-TOF 384-well plate for MALDI TOF/TOF analysis.

### MALDI-TOF MS analysis of peptides

Spectra were acquired on a 4800 MALDI TOF/TOF mass spectrometer (Sciex) fitted with a UV laser (wavelength: 337 nm) in reflectron mode. All MS spectra of the tryptic digests were externally calibrated using a mixture of peptide standards. MS/MS spectra were acquired on selected ions of interest under the following conditions: precursor isolation set to resolution of 200, collision energy of 2 kV, CID cell pressure of 2 × 10–5 torr, air as collision gas. The instrument was calibrated in MS/MS mode using five daughter ions (m/z: 175.119, 684.346, 813.389, 1056.475 and 1441.634) generated from the fragmentation of Glu-fibrino peptide (m/z: 1570.6774). Data were analysed comparing the monoisotopic peaks with the theoretical values corresponding to the expected peptide digestion products. Peak lists were searched with Mascot Server using the following parameters: enzyme = trypsin; maximum missed cleavages = 2; variable modifications = carbamidomethylation of cysteine, oxidation of methionine; mass tolerance = 50 ppm. The SwissProt database (homo sapiens, human) was used as reference protein sequence database.

### Statistical Analyses

All experimental results are expressed as mean ± SD. Statistical significance was assessed by one-way ANOVA with Dunnett’s post test and was performed using Prism version 6.01 (GraphPad Software, Inc.).

### Protein Preparation

The monomer A (chain A) of hMGL crystallized in its open conformation (PDB: 3HJU)^8^ was used. The protein was prepared using the Protein Preparation Wizard workflow within Maestro 9.6 in the Schrödinger 2013–3 suite^34^. Hydrogen atoms were added to the structure and the protonation state of ionisable amino acids were chosen to be consistent with physiological pH. The overall hydrogen bonding network was optimized by fixing the tautomers of histidine residues and the orientation of hydroxyl and thiol groups, as well as the orientation of asparagine and glutamine side chains. Protein *termini* were protected with capping neutral groups. The glycerol molecule co-crystallized within MGL active site was removed, and the system was subjected to a restrained minimization using the OPLS2005 force field^35^ to an overall RMSD of 0.3 Å. Models of hMGL with modifications at C201 and C208 side chains (sulfenic acid, sulfenamide and site directed mutations to alanine) were built in Maestro 9.6.

### Molecular Dynamics systems preparation

Molecular Dynamics Simulations were performed using Desmond 3.6 implemented within the Schrödinger 2013–3 suite (Maestro-Desmond Interoperability Tools, version 3.6)^36^. For the MD simulations in water, the protein was solvated by approximatively 30600 spc water molecules^37^ in a simulation box of 76 Å × 72 Å × 64 Å. The net charge of the system was neutralised by adding one Na^+^ atom. For simulations of the enzyme in presence of the membrane, the hMGL structure was embedded in a POPC lipid bilayer model according to the coordinates of the 3HJU crystal structure deposited into the Orientation of Protein in Membrane (OPM) database^38^. The protein-membrane complex was then solvated by approximatively 63000 spc water molecules, in a simulation box of 84 Å × 81 Å × 136 Å. The net charge of the system was neutralised by adding one Na^+^ atom. The OPLS2005 force field implemented in Desmond was used to model the systems. Bond lengths to hydrogen atoms were constrained by applying the M-SHAKE algorithm. Short-range electrostatic interactions were cut off at 9 Å, whereas long-range electrostatic interactions were treated using the Smooth Particle Mesh Ewald method. A RESPA integrator was used with a time-step of 2 fs, while long-range electrostatic interactions were computed every 6 fs. Each MD simulation was carried out in the NPT ensemble, coupling to the Langevin thermostat method. A detailed description of the equilibration protocol is provided in the Supplementary Information.

### Well-Tempered Metadynamics parameters

The system was prepared following the same procedure used for the MD simulations. The free-energy profile of native hMGL was computed using the Well-Tempered Metadynamics implemented in Desmond 3.6. In our simulations, distances between centres of mass of Cα of selected residues on the lid domain were used as CV1 (distance between 176–179 Cα and 150–157 Cα centres of mass) and as CV2 (distance between 176–179 Cα and 158–164 Cα centres of mass). The Gaussian width was set to 0.05 Å, the Gaussian height was set to 0.03 kcal/mol, while the deposition time was 0.09 ps. In WT Metadynamics, the Gaussians heights *w_j_* are resized taking into account the value of accumulated bias potential *V(s*, *t)*:


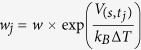


where the initial height of the Gaussians *w* was 0.03 kcal/mol, *k*_*B*_ is the Boltzmann constant and the sampling temperature *ΔT* was 1200 K. Each WT Metadynamics simulation lasted for 100 ns and was replicated twice, changing the seed to assign starting velocities. The evaluation of convergence for WT Metadynamics simulations was based on three criteria. 1) Throughout the simulation, we compared the FESs updated at intervals of 5 ns and, to consider a simulation to be converged, we checked that the free-energy profiles did not change significantly. This was achieved both comparing the FESs updated at different simulation times (Fig. S4) and checking the values of free-energy differences between each minimum and the global minimum (Fig. S5). 2) We checked that each collective variable had been completely explored with at least one recrossing, to avoid stopping the simulation with the system trapped in a specific free-energy minimum (Fig. S6a,b). The evolution of the height of the Gaussians deposited throughout the WT Metadynamics simulation was also registered (Fig. S6c). The sequence of damping and increasing phases indicates that depositions explored different minima several times. 3) We checked that, at convergence, the FES profiles of different replicas were superposable (Fig. S7).

## Additional Information

**How to cite this article**: Scalvini, L. *et al.* Free-energy studies reveal a possible mechanism for oxidation-dependent inhibition of MGL. *Sci. Rep.* **6**, 31046; doi: 10.1038/srep31046 (2016).

## Supplementary Material

Supplementary Information

## Figures and Tables

**Figure 1:**
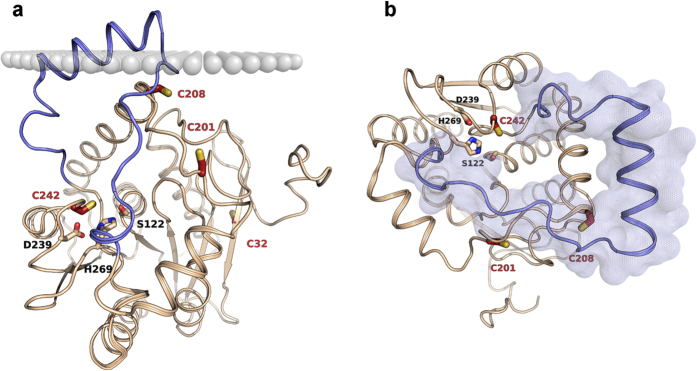
Crystal structure of hMGL with the lid domain in its open conformation (PDB 3HJU) represented in beige cartoon. The upper portion of the lid domain, delimiting the access to the catalytic site, is represented in light blue. The catalytic triad (S122, H269 and D239) are represented in sticks with beige carbons, while regulatory cysteine residues (C201, C208, C242) are depicted in bold sticks with red carbons. A putative membrane profile is represented with grey spheres (**a**). The lid domain stands above the active site, delimiting a U-shaped access funnel (**b**).

**Table 1:** MALDI TOF MS Fingerprinting of Cys-containing peptides in control hMGL.

Start-End	Observed [M+H]^+^	Calc. [M+H]^+^	Error (ppm)	MC	Peptide	Modif.	S/N
10–33	2832.3942	2832.3773	5.97	0	TPQSIPYQDLPHLVNADGQYLF**C**R	CAM	146
187–202	1936.9514	1936.9433	4.18	1	NKTEVDIYNSDPLI**C**R	CAM	124
189–202	1694.8107	1694.8054	3.13	0	TEVDIYNSDPLI**C**R	CAM	93
207–219	1476.8049	1476.7991	3.53	0	V**C**FGIQLLNAVSR	CAM	164
241–255	1711.8302	1711.8757	−26.6	1	L**C**DSKGAYLLMELAK	CAM	7

**Table 2:** MALDI TOF MS Fingerprinting of Cys-containing peptides in hMGL incubated with H_2_O_2_ and DMD.

Start-End	Observed [M+H]^+^	Calculated [M+H]^+^	Error (ppm)	Peptide	Modif.	S/N
10–33	2832.3943	2832.3773	6.00	TPQSIPYQDLPHLVNADGQYLF**C**R	CAM	20
187–202	1936.9464	1936.9433	1.60	NKTEVDIYNSDPLI**C**R	CAM	68
187–202	2018.0080	2017.9983	9.27	NKTEVDIYNSDPLI**C**R	DMD	10
189–202	1694.7904	1694.8054	−8.85	TEVDIYNSDPLI**C**R	CAM	39
189–202	1775.8528	1775.8514	0.79	TEVDIYNSDPLI**C**R	DMD	7
207–219	1476.7851	1476.7991	−9.5	V**C**FGIQLLNAVSR	CAM	64
241–255	1711.8450	1711.8684	−17.9	L**C**DSKGAYLLMELAK	CAM	4

**Table 3:** MALDI TOF/TOF fragmentation of C201-containing tryptic peptide NKTEVDIYNSDPLICR (187–202).

Observed [M+H]^+^	Modification	y-5 Fragment	Observed m/z fragment	Calculated m/z fragment	Error (ppm)
1879.7920	None	PLICR	601.3415	601.3490	−12.5
1936.8920	CAM	PLICR	658.3679	658.3705	−3.95
2017.9983	DMD	PLICR	739.4206	739.4170	4.87

Related MS/MS spectra are reported in the Supplementary Information (Fig. S2).
