# Supplementary material for: Free-energy studies reveal a possible mechanism for oxidation-dependent inhibition of MGL
Source: Sci Rep. 2016 Aug 8;6:31046. doi: 10.1038/srep31046 (PMC4976315; doi:10.1038/srep31046)
Supplement: Supplementary Information [file srep31046-s1.pdf]

Supplementary Information

Free-energy studies reveal a possible mechanism for oxidation-dependent inhibition of MGL

Laura Scalvini[1], Federica Vacondio[1], Michele Bassi[1], Daniele Pala[1], Alessio Lodola[1], Silvia Rivara[1], Kwang-Mook Jung[2], Daniele Piomelli[2,3,4], Marco Mor[1*]

[1] Dipartimento di Farmacia, Università degli Studi di Parma, I-43124 Parma, Italy

[2] Department of Anatomy and Neurobiology, University of California, Irvine, Irvine, CA 92697, United States

[3] Department of Biological Chemistry, University of California, Irvine, Irvine, CA 92697, United States

[4] Unit of Drug Discovery and Development, Istituto Italiano di Tecnologia, I-16163, Genova, Italy

* Corresponding author:

Marco Mor

Dipartimento di Farmacia, Università degli Studi di Parma

Area delle Scienze 27/A

I-43124 Parma, Italy

marco.mor@unipr.it

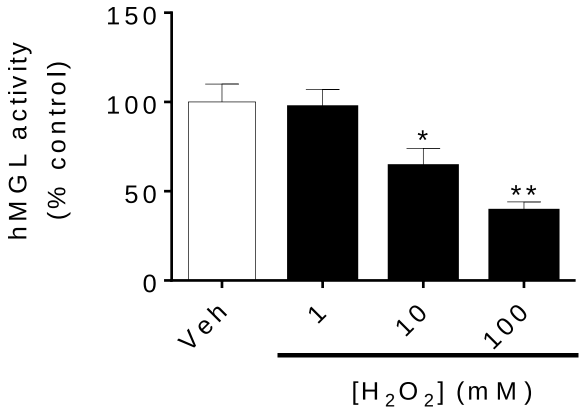

Figure S1. H_2O_2 inactivates hMGL Effects of increasing concentrations of H_2O_2 on hMGL basal activity. hMGL activity in the presence of vehicle was set as 100%. Error bars represent SD (n=3). $^{**}P < 0.005$ and $^{*}P < 0.05$ compared with vehicle, one-way ANOVA.

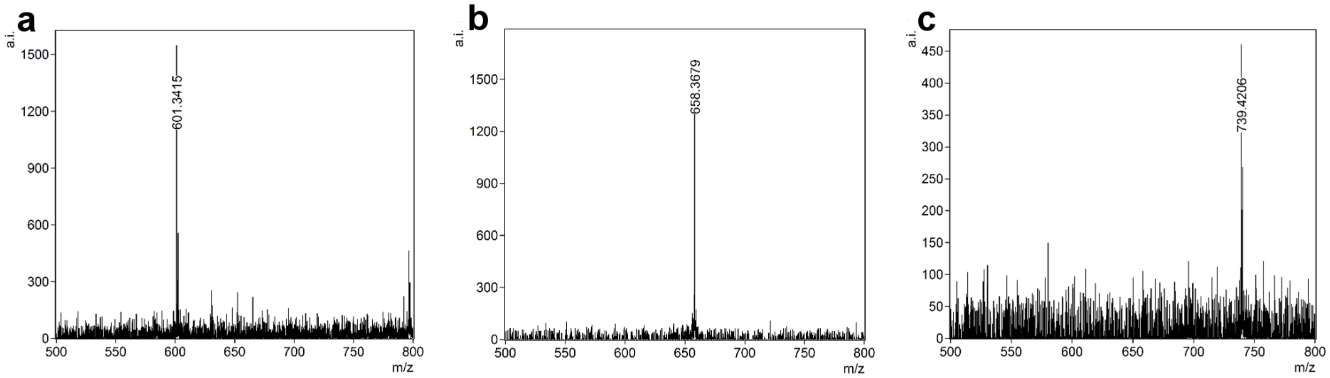

Figure S2, related to Table 3. MS/MS spectra showing the y-5 fragment under different conditions. a) Native y-5 fragment; **b**) CAM-modified y-5 fragment and **c**) DMD-modified peptide.

Supplementary Computational Procedure

Equilibration protocol of Molecular Dynamics and Metadynamics simulations performed in water

Each simulation performed was carried out in the NPT ensemble, coupling to the Langevin thermostat method, at a temperature of 300 K. Each system was previously equilibrated by applying the following protocol:

1. Steepest descent energy minimization with the solute heavy atoms restrained
2. Steepest descent energy minimization without any restraints
3. 200 ps simulation in the NVT ensemble, coupling to the Langevin thermostat with:
    a. Simulation temperature of 10 K
    b. 50 kcal mol^{-1} $Å^{-2}$ restraints on solute heavy atoms
4. 100 ps simulation in the NVT ensemble using the Langevin thermostat with:

    a. Simulation temperature of 100 K
    b. 25 kcal mol^{-1} $Å^{-2}$ restraints on protein backbone and beta carbons
5. 100 ps simulation in the NVT ensemble, using the Langevin thermostat with:
    a. Simulation temperature of 200 K
    b. 25 kcal mol^{-1} $Å^{-2}$ restraints on protein backbone and beta carbons
6. 400 ps simulation in the NVT ensemble, using the Langevin thermostat with:
    a. Simulation temperature of 300 K
    b. 12.5 kcal mol^{-1} $Å^{-2}$ restraints on protein backbone and beta carbons
7. 400 ps simulation in the NPT ensemble, using the Langevin thermostat with:
    a. Simulation temperature of 300 K
    b. 12.5 kcal mol^{-1} $Å^{-2}$ restraints on protein backbone
8. 800 ps simulation in the NPT ensemble, using the Langevin thermostat with:
    a. Simulation temperature of 300 K
    b. 5 kcal mol^{-1} $Å^{-2}$ restraints on protein backbone
9. 1000 ps simulation in the NPT ensemble, using the Langevin thermostat with:
    a. Simulation temperature of 300 K
    b. 5 kcal mol^{-1} $Å^{-2}$ restraints on protein backbone, excluding residues 150-173 (residues of the helix α4)
10. 1000 ps simulation in NPT ensemble, using the Langevin thermostat with:
    a. Simulation temperature of 300 K
    b. No restraints

Equilibration protocol of Molecular Dynamics and Metadynamics simulations in presence of the membrane model

Each simulation performed was carried out in the NPT ensemble, coupling to the Langevin thermostat method, at a temperature of 300 K. Each system was previously equilibrated by applying the following protocol:

1. Steepest descent energy minimization with the solute heavy atoms restrained
2. Steepest descent energy minimization without any restraints
    - Membrane relaxation protocol
        1. 4000 ps simulation in the NVT ensemble, using the Berendsen thermostat/barostat coupling method with:
            a. Simulation temperature progressively rising from 0 to 300 K
            b. 50 kcal mol^{-1} $Å^{-2}$ restraints on solute heavy atoms
        2. 2000 ps simulation in the NPT ensemble, using the Berendsen thermostat/barostat coupling method with:
            a. Simulation temperature of 300 K
            b. 50 kcal mol^{-1} $Å^{-2}$ restraints on solute heavy atoms
        3. 2400 ps simulation in the NPT ensemble, using the Berendsen thermostat/barostat coupling method with:
            a. Simulation temperature of 300 K

    b. Restraints progressively reducing from 50 to 10 kcal mol^{-1} $Å^{-2}$ on solute heavy atoms.

4. 2000 ps simulation in the NPT ensemble, using the Berendsen thermostat/barostat coupling method with:
    a. Simulation temperature of 300K
    b. Restraints progressively reducing from 10 to 5 kcal mol^{-1} $Å^{-2}$ on solute heavy atoms.
5. 1000 ps simulation in the NPT ensemble, using the Berendsen thermostat/barostat coupling method with:
    a. Simulation temperature of 300K
    b. 5 kcal mol^{-1} $Å^{-2}$ restraints on the protein backbone

After the membrane equilibration, a 3000 ps system equilibration stage was performed, using the protocol used for the simulations in water.

a

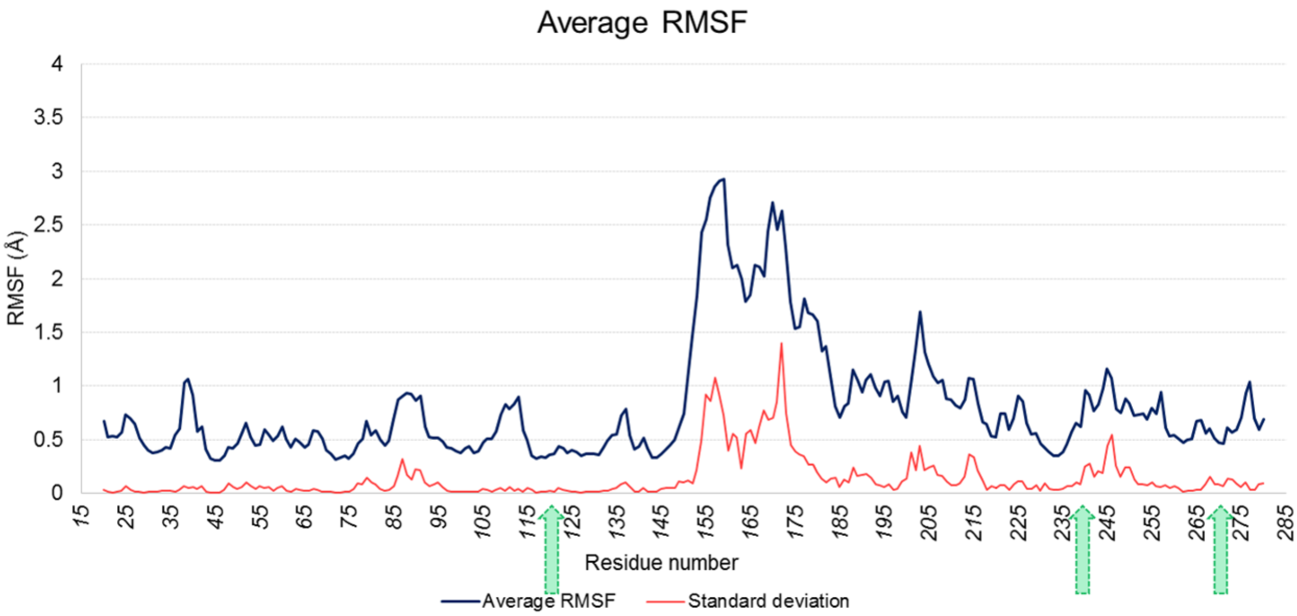

b

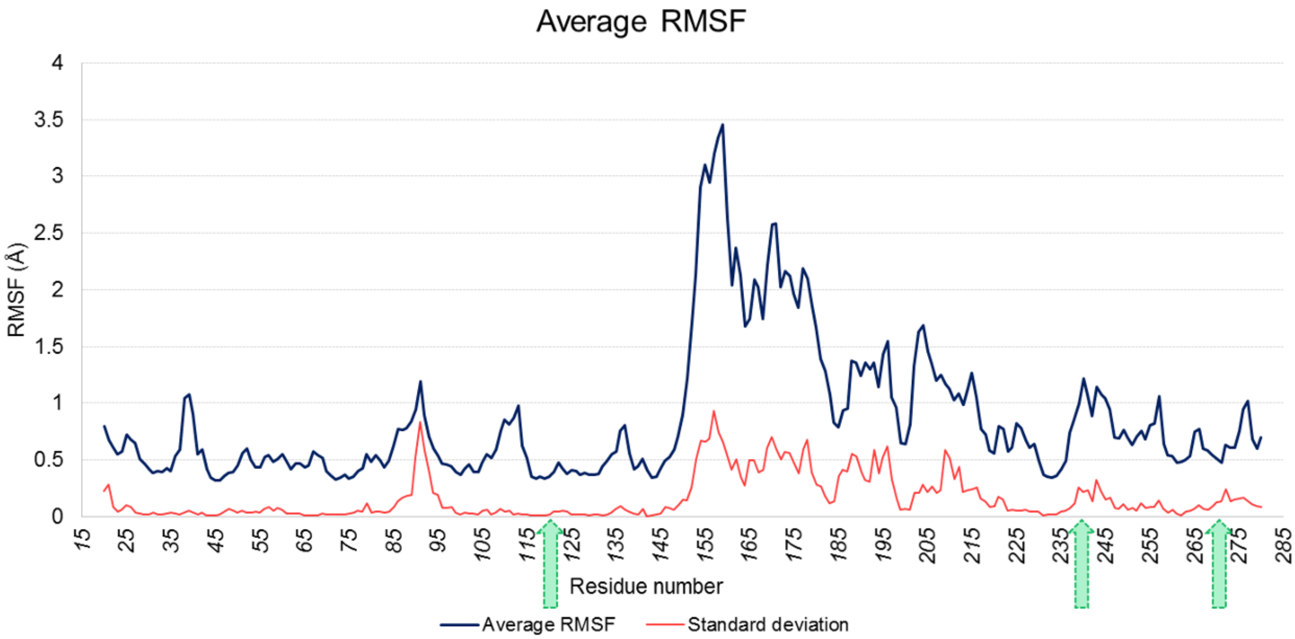

Figure S3. RMSF analysis of MD simulations performed in water of native hMGL (a) and of hMGL modelled with C201 mutated to sulfenic acid (b). The average RMSF value (blue line) was calculated for the backbone of residues 20-280. The analysis was carried out taking into account all the MD simulations, and an average structure from each set of simulations was used as reference structure. The standard deviation value is reported (red line). While the β core of the protein maintains a stable conformation, residues 150-215, which comprise the lid domain, are characterised by higher flexibility. Green arrows mark the position of the catalytic triad residues (S122, H269 and D239).

Convergence of WT Metadynamics simulations

The evaluation of convergence for a free-energy surface (FES) calculated by a WT Metadynamics simulation was based on three criteria. The following figures report the results of a representative simulation, with wild-tipe MGL and model membrane.

1) Throughout the simulation, we compared the FESs updated at intervals of 5 ns and, to consider a simulation to be converged, we checked that the free-energy profiles did not change significantly. This was achieved both comparing the FESs updated at different simulation times (Fig. S4) and checking the values of free-energy differences between each minimum and the global minimum (Fig. S5).

2) We checked that each collective variable (CV) had been completely explored with at least one recrossing, to avoid stopping the simulation with the system trapped in a specific free-energy minimum (Fig. S6a-b). The evolution of the height of the Gaussians deposited through the WT Metadynamics simulation was also registered (Fig. S6c). The sequence of damping and increasing phases indicates that depositions explored different minima several times.

3) We replicated WT Metadynamics simulations changing the seed to calculate initial velocities. At convergence, the FES profiles were reproducible (Fig. S7).

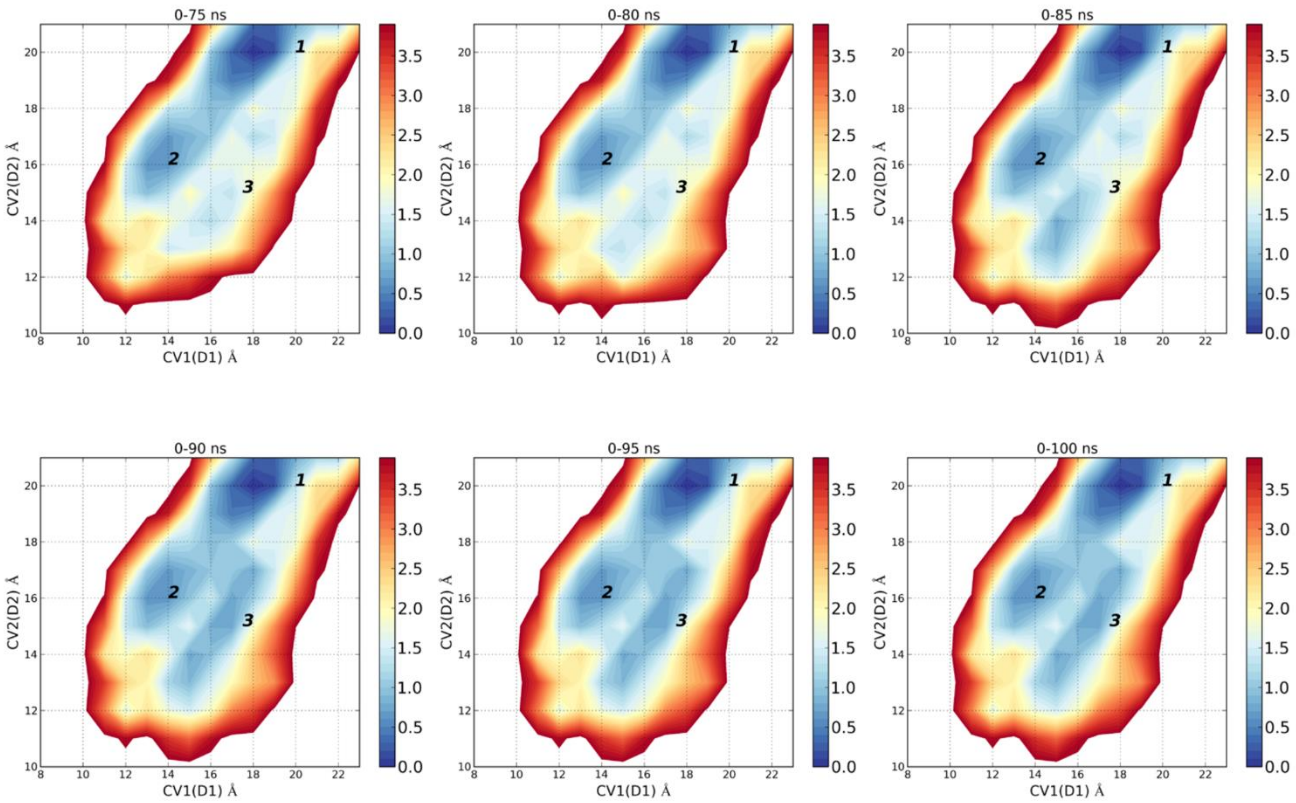

Figure S4. Evolution of the free energy surfaces during the last 25 ns of 100 ns WT Metadynamics of native hMGL, in the presence of a membrane model, projected along CV1 and CV2. The FES obtained after 100 ns of WT Metadynamics corresponds to Figure 5b in the main text) Numbers 1, 2 and 3 mark the global and local minima on the FESs.

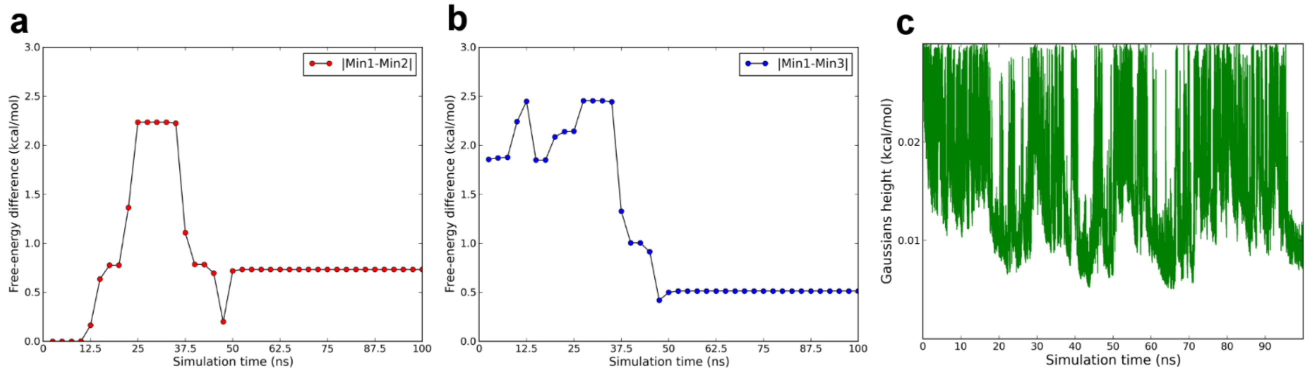

Figure S5 Evolution of free-energy differences among a) minimum 1 (global minimum, see Fig. S4) and 2, b) and minimum 1 and 3, at different simulation times. The evolution of the height of the Gaussians deposited through all the WT Metadynamics simulation is also reported (c).

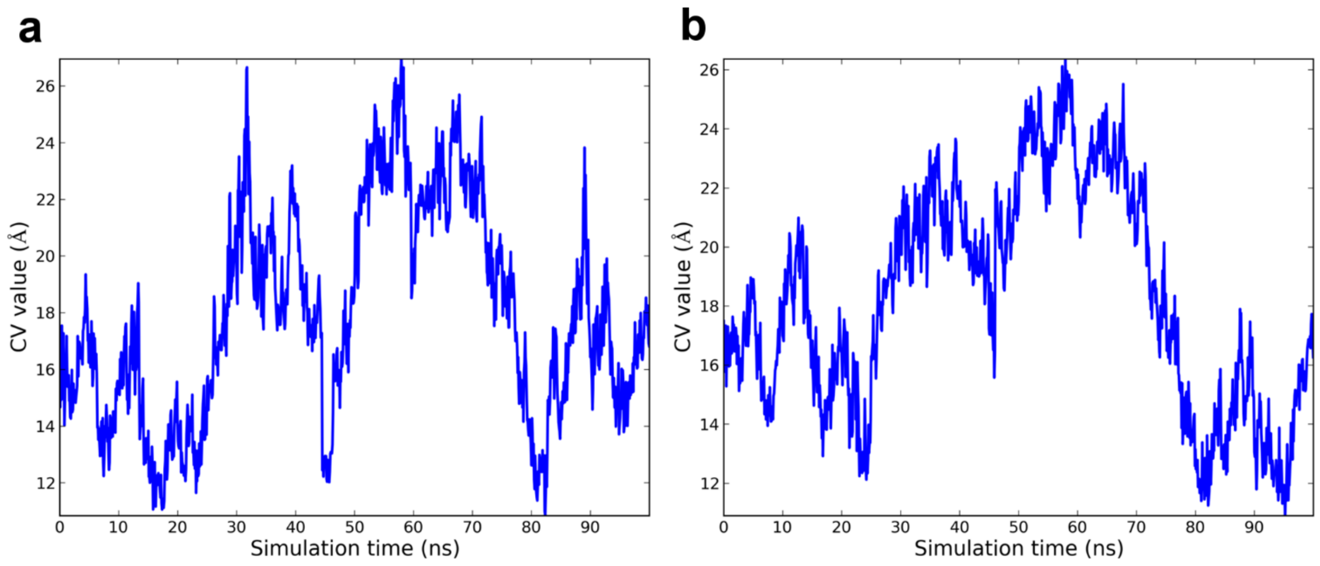

Figure S6 Evaluation of the diffusive behaviour of CV1 (a) and CV2 (b) throughout the WT Metadynamics simulation.

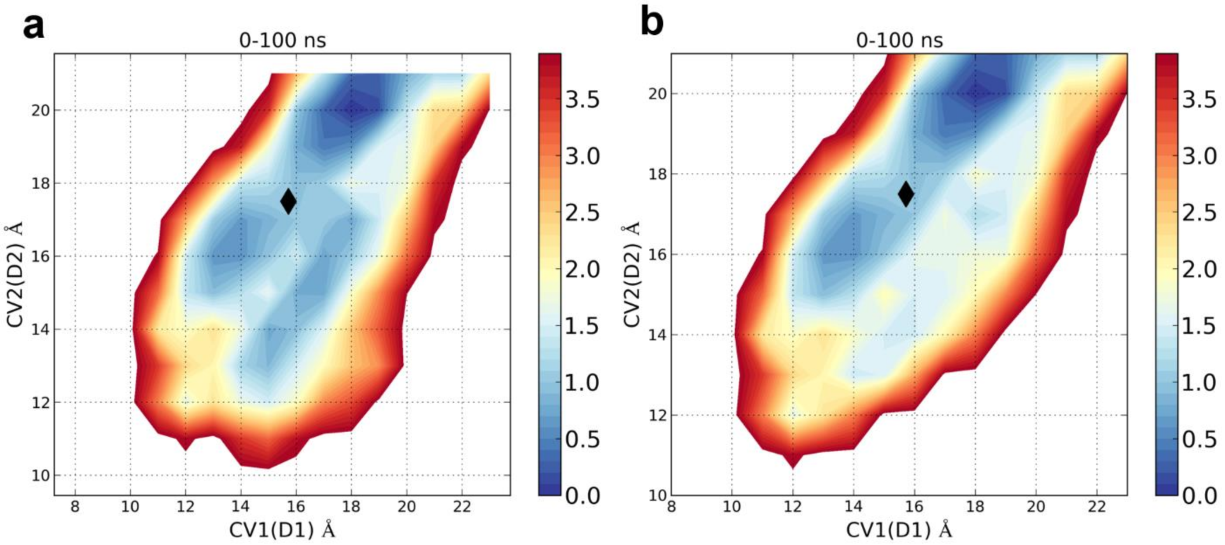

Figure S7 Comparison of FES obtained for two independent WT Metadynamics simulations of native hMGL modelled in presence of a the membrane. Seeds for calcualtion of initial velocities were different in the two simulations. A black diamond marks the value of CV1 and CV2 measured in the crystal structure 3HJU. Panel **a** corresponds to Figure 5b in the main text.
